# Reward sensitivity and action in Parkinson’s disease patients with and without apathy

**DOI:** 10.1093/braincomms/fcab022

**Published:** 2021-03-10

**Authors:** Kinan Muhammed, Michael Ben Yehuda, Daniel Drew, Sanjay Manohar, Masud Husain

**Affiliations:** Nuffield Department of Clinical Neurosciences, University of Oxford, Oxford OX3 9DU, UK; Department of Experimental Psychology, University of Oxford, Oxford OX2 6GG, UK; Department of Experimental Psychology, University of Oxford, Oxford OX2 6GG, UK; Nuffield Department of Clinical Neurosciences, University of Oxford, Oxford OX3 9DU, UK; Department of Experimental Psychology, University of Oxford, Oxford OX2 6GG, UK; Nuffield Department of Clinical Neurosciences, University of Oxford, Oxford OX3 9DU, UK; Department of Experimental Psychology, University of Oxford, Oxford OX2 6GG, UK; Nuffield Department of Clinical Neurosciences, University of Oxford, Oxford OX3 9DU, UK; Department of Experimental Psychology, University of Oxford, Oxford OX2 6GG, UK

**Keywords:** Parkinson’s disease, apathy, dopamine, reward sensitivity, pupillometry

## Abstract

Clinical apathy results in dysfunction of goal directed behaviour, a key component of which is the initiation of action. Previous work has suggested that blunting of reward sensitivity is an important mechanism underlying apathy. However, an additional component might be impoverished initiation of action itself. This study aims to investigate the link between motivation and motor output and its association with apathy and dopamine. An oculomotor task that measures pupillary and saccadic response to monetary incentives was used to assess reward sensitivity, first in 23 young and 18 elderly controls, and then in 22 patients with Parkinson’s disease tested ON and OFF dopaminergic medication. To distinguish between pupillary responses to anticipated reward alone versus responses associated with motor preparation, a saccadic ‘go/no-go’ task was performed. Half of the trials required a saccade to be initiated to receive a reward and in the remaining trials no action was required but reward was still obtained. No significant difference in pupil response was demonstrated between the two conditions in all groups tested, suggesting pupillary responses to rewards are not contingent upon motor preparation in Parkinson’s disease. Being ON or OFF dopamine did not influence this response either. Previous work demonstrated associations between apathy and pupillary reward insensitivity in Parkinson’s disease. Here we observed this effect only when an action was required to receive a reward, and only in the ON state. These findings suggest that apathy in Parkinson’s disease is linked to reduced reward sensitivity and that this is most prominently observed when actions have to be initiated to rewarding goals, with the effect modulated by being ON dopaminergic medication. OFF medication, there was no such strong relationship, and similarly in the ‘no-go’ conditions, either ON or OFF dopaminergic drugs. The results provide preliminary data which suggest that apathy in Parkinson’s disease is associated with a reduction in reward sensitivity and this is most evident when associated with initiation of goal directed actions in the presence of adequate dopamine.

## Teaser

Muhammed *et al*. investigate the link between motivation and motor output and its association with apathy and dopamine in patients with Parkinson’s disease and healthy controls. Using an oculomotor reward task, links between pupillary responses to rewards and motor action in differing dopaminergic states was explored.

## Graphical Abstract

**Figure:**
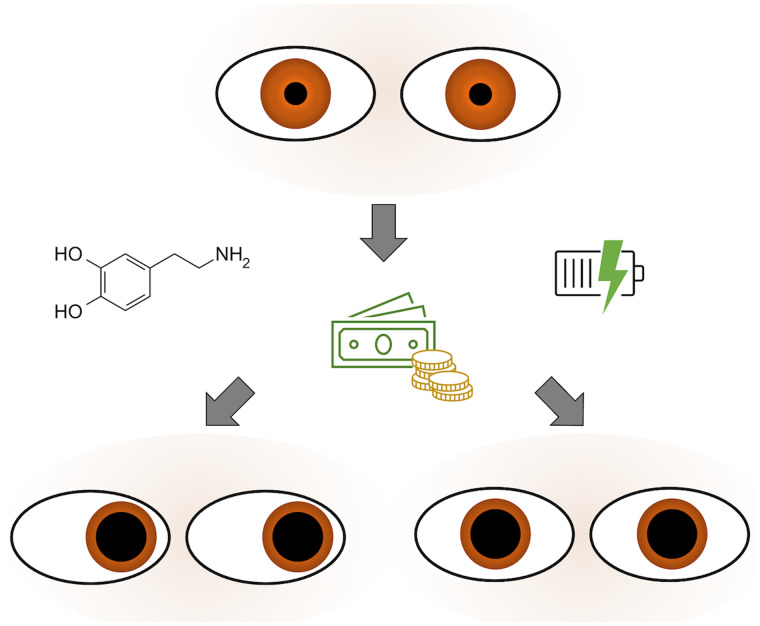

## Introduction

Clinical apathy is a syndrome which manifests as a reduction in goal directed behaviour and is associated with significant reduction in quality of life.^1–6^ It is common in neurodegenerative disorders, particularly Parkinson’s disease with a prevalence ranging up to 70%.^7–12^ Importantly, apathy is considered not to be a secondary psychological reaction to physical impairments of Parkinson’s disease,^8^ but instead a consequence of neurodegeneration of frontostriatal regions, including reward sensitive regions of the basal ganglia, such as the ventral striatum, and the dorsal anterior cingulate cortex—areas that play a central role in motivated behaviour and initiation of actions.^13^

Over the last few years, several studies have suggested blunted reward sensitivity forms an important component of apathy. Most notably, the association with reward sensitivity and apathy has been demonstrated in Parkinson’s disease and found to be modulated by dopamine. This has been explored using different behavioural paradigms, including effort-based decision-making tasks and oculomotor eye tracking experiments.^14–17^ Results from reward-effort discounting assessments demonstrate Parkinson’s disease patients with apathy are less inclined to make a physical effort for reward compared to those without apathy, particularly if the reward level is low.^17^ Additionally, dopamine therapy in these patients increases both motor vigour and engagement rate for high effort and higher offer rewards, highlighting dopamine’s modulatory effect.

Similarly, the ability to objectively measure reward sensitivity has also been demonstrated in oculomotor experiments.^18–22^ On anticipation of a reward, pupil dilatation in healthy individuals and those with Parkinson’s disease, scales with incentives on offer prior to making eye movements to obtain them. This anticipatory pupil response is blunted in patients with clinical apathy and again modulated by dopamine, with dopaminergic medication increasing reward sensitivity.^16^ Dopamine’s modulatory effect on reward sensitivity makes it an important neurotransmitter in motivational processes that underpin goal-directed behaviour.^23–28^ In rodents, dopamine signals in the ventral striatum ramp up with increasing proximity to and value of future rewards as animals engage in physical effort to reach them.^29^ In humans, focal lesions of the basal ganglia can lead to apathy, which may be reversed by dopaminergic medication in some cases.^26^^,^^30^

Most behavioural tasks that attempt to assess associations with reward sensitivity, apathy and Parkinson’s disease, require motor acts to be performed in order to obtain a reward. Indeed, a proposed mechanism of goal directed behaviour is the requirement to initiate actions to achieve an objective.^7^^,^^31^ But is reward sensitivity dependent on goal directed action—the motor response normally required to obtain a rewarding goal—or does it occur regardless of motor preparation? One possible interpretation of the findings in Parkinson’s disease patients is that that those with clinical apathy have dysfunction in their evaluation of rewards and this might explain the paucity of their actions, a key clinical characteristic of the syndrome.^32^ But impairments in the process of translating valuation into action, in addition simply to reward sensitivity, might also be important in apathy but this has yet to be fully explored in patients.

Imaging studies in humans reliably identify ventral frontal and striatal areas in association with apathy.^13^^,^^33^ In healthy young people, apathy correlates with reduced white matter connectivity between rostral cingulate areas and medial brain regions linked to movement control including the supplementary motor area.^24^ The same apathetic participants also appeared to incur larger costs (increased brain activation) in initiating effort. Therefore, apathy may encompass difficulty in the transformation of an intention (motivation to act) to the action itself, possibly via limbic-motor system dysfunction.^7^^,^^34^

In animals, dopamine has been specifically implicated in the transition from incentive evaluation to initiation of action. Dopamine within the nucleus accumbens (NAcc) of the ventral striatum, increases when a cue for a reward is heard and, crucially, a movement *also must be initiated* in order to receive the reward.^35^ This phasic dopamine response peaks just before receipt of a reward and is not present when a movement is not required to obtain it, even though the reward cue conveyed the same information about the upcoming incentive. Dopamine release in the NAcc therefore appears *contingent on initiation of motor actions* and not just reward evaluation, implicating it as a potential interface between limbic and motor systems.^36^^,^^37^ Indeed, lesions to limbic regions alter evaluation of reward and effort costs.^38^ Damage to either the ACC or mesolimbic dopamine pathways in rats and monkeys, creates a bias in preference towards options that require low effort but for relatively small reward,^28^^,^^39^ resulting in devaluation of reward and increased sensitivity to the effort costs for actions. The NAcc receives dopaminergic inputs from the ventral tegmental area (VTA) and degeneration of the VTA-NAcc pathway in 1-methyl-4-phenyl-1,2,3,6-tetrahydropyridine (MPTP) lesioned monkeys has specifically been associated with apathy.^40^

These considerations suggest that attempts to probe the complex interplay between brain regions associated with reward valuation and initiation of movements may enhance understanding of mechanisms underlying apathy in neurodegenerative diseases such as Parkinson’s disease. In a previous oculomotor study, apathetic Parkinson’s disease patients demonstrated reduced pupillary response to incentives, modulated by dopaminergic state, being blunted when patients were OFF dopaminergic drugs.^16^ Crucially, however, it was not established whether the observed pupillary reward sensitivity effects were contingent on action, or to dopaminergic status when an action was *not performed*. Nor was it determined how this might relate to apathy.

Therefore, in this study, we aim to investigate the relationship between reward evaluation and movement initiation in healthy participants and those with Parkinson’s disease, focusing on specific oculomotor and pupillary responses to reward, the effect of dopamine and the link to apathy. We aim to address the following questions: First, do anticipatory pupil responses to rewards arise due to motor preparation signals linked to performing an action, or are such anticipatory pupil responses to reward dissociable from movement? Second, are reward sensitivity deficits in Parkinson’s disease patients with clinical apathy linked to impairments in the evaluation of rewards only or, are the initiation of actions also crucial for association of reward cues? Finally, how are anticipatory pupillary responses associated with or without actions, modulated by dopaminergic state in Parkinson’s disease?

In order to address these questions, an adaptation of a previous oculomotor study ^16^ was performed. This was carried out in young and elderly healthy participants as well as a group of patients with idiopathic Parkinson’s disease, assessed both ON their normal dopaminergic medication and while OFF. The task was a novel variation of a ‘go/no-go’ paradigm designed specifically to explore initiation of actions rather than response inhibition as no reflexive movements needed to be suppressed. It comprised, within the same block of trials, two separate types of trial, one where a motor action (saccadic eye movement) was made to receive a monetary reward, and another where no action was required for reward. Saccadic eye movement parameters and pupillary response to reward cues were measured in all the participant groups, as well as being related to clinical apathy, and being ON or OFF dopaminergic drugs, in Parkinson’s disease patients.

## Materials and methods

### Participants

Healthy young and elderly people and patients with idiopathic Parkinson’s disease were recruited. The study was approved by the local ethics committee, written consent was obtained from all subjects, and the protocol followed the principles of the Declaration of Helsinki. Young participants were enlisted through the online recruiting system of the Department of Experimental Psychology at the University of Oxford. Elderly control subjects were sampled from a pool of volunteers registered with the Oxford Dementia and Ageing Research (OxDARE). Parkinson’s disease patients were recruited from clinics in the Oxfordshire area. A minimum of £8 and maximum of £12 was paid for participation in the study and transport costs were reimbursed. Parkinson’s disease patients were assessed for apathy using the Lille Apathy Rating Scale (LARS)^41^; they were also screened for depression with the Beck Depression Inventory-II (BDI-II)^42^ and for cognitive impairment using the Montreal Cognitive Assessment (MoCA).^43^ Parkinson’s disease severity was measured using the Movement Disorder Society Unified Parkinson's disease rating scale (UPDRS)^44^ and Hoehn & Yahr Staging. Levodopa equivalent doses were calculated for all Parkinson’s disease patients. All participants had normal or corrected to normal vision at the time of testing and neurological examination revealed no oculomotor deficits.

### Demographics

Twenty-three young healthy volunteers and eighteen elderly control participants with no history of psychiatric or neurological disorders took part in the experiment [Young: mean age 23.8 years (SD ±5) 7 male; Elderly: mean age 70.4 years (SD ±6), 11 male]. Twenty-two patients with idiopathic Parkinson’s disease were recruited, all were screened for any pre-existing psychiatric disorders and any significant comorbidities. Average age 65.2 (SD ±8.9) and 14 males. Nine patients were on levodopa only, one on dopamine agonist only and the remaining 12 were on a combination of both. Average disease duration 5 years (SD ±4). Eight patients scored worse than the −22 clinical apathy threshold on the LARS, see Table 1 for full demographic breakdown. The UPDRS III was performed when the patients were in the ON state only. LARS, MoCA and BDI-II were completed for Parkinson’s disease patients and elderly controls to allow direct comparisons between these two groups. Numbers of participants are typical of similar studies in the field.^16^

**Table 1:** Demographic comparison of Parkinson’s disease patients and elderly controls

	Healthy elderly controls	Parkinson’s disease (medicated)	Elderly controls versus Parkinson’s disease *P*-value
*n*	18	22	
Age (years)	70.4 (±6)	65.2 (±8.9)	0.03^a^
Apathy (LARS)	−26 (±5.4)	−23.5 (±8.5)	0.2
Depression Score (BDI-II)	5.4 (±4.0)	11.3 (±7.0)	<0.01^a^
MoCA	27.5 (±2.2)	28.6 (±1.4)	0.06
UPDRS I	NA	5.5 (±4.1)	NA
UPDRS III ON	NA	18.5 (±9.8)	NA
Hoehn and Yahr Stage	NA	1.4 (±0.6)	NA
Hours since last dose ON versus OFF	NA	2.4 (±2.4) versus 14.1 (±4.1)	NA
Levodopa equivalent dose (mg/24 h)	NA	615 (±72.7)	NA

Numbers in brackets represent standard deviations and standard error of the mean where appropriate.

Eight Parkinson’s disease patients and three controls scored worse than the −22 apathy threshold on the LARS, 1 Parkinson’s disease patient was classified as having moderate depression (21–30 on the BDI-II), 4 borderline (17–20 on the BDI-II), and the remainder fell below the depression classification threshold (<16 on the BDI-II).

^a^Indicates a significant difference.

BDI-II = Beck Depression Inventory II; LARS = Lille Apathy Rating Scale; MoCA = Montreal Cognitive Assessment; UPDRS = Unified Parkinson’s disease rating scale.

### Experimental paradigm

**Figure 1:**
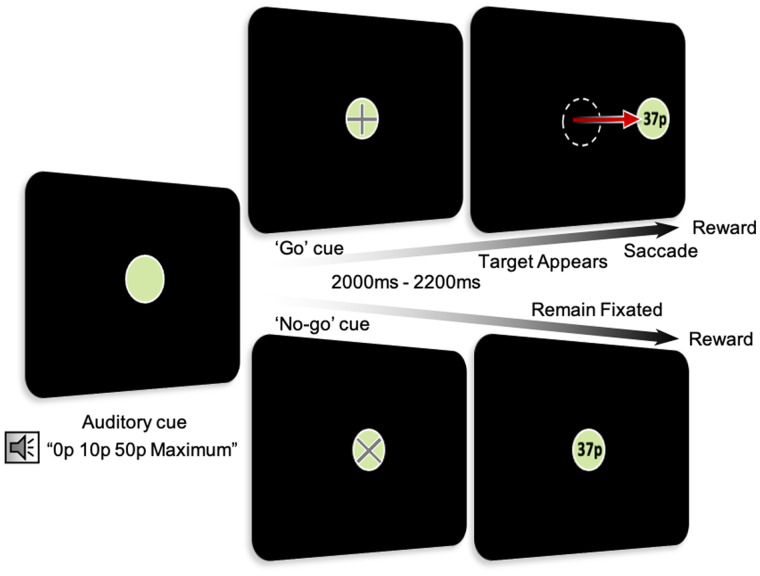
**Oculomotor experimental paradigm.** During the task, 50% of trials required a saccade to be made to obtain a reward (‘go’ trials indicated by a ‘+’ at the start of the trial). In the remaining 50% of trials, participants were required to maintain fixation centrally (‘no-go’ trials indicated by an ‘x’ at the start of the trial). ‘go’ and ‘no-go’ trials were randomly intermixed. Reward obtained was based on reaction time in the ‘go’ trials and reward rates in both arms were matched.

The task design was adapted from a previous study.^16^ Participants were instructed that the quicker they looked at a target, the greater the proportion of reward on offer they would obtain (Fig. 1). Each trial commenced with the onset of a disc at screen centre. After they had fixated this for 500 ms, participants heard a recorded voice that informed them of the maximum reward available for that particular trial: ‘0p/10p/50p maximum’. Simultaneously with the auditory reward cue either a ‘+’ or a ‘×’ symbol would appear within the fixation target for 200 ms. The ‘+’ indicated a ‘go’ trial and the ‘×’ a ‘no-go’ trial. ‘×’ and ‘+’ symbols were used as visual cues because they were the same graphical image rotated 45°. This allowed luminance of the visual cue to be matched and direct comparisons to be made.

#### ‘Go’ trials

A go trial was indicated by the appearance of a ‘+’ symbol. After a randomly variable period of 2000, 2100 or 2200 ms the central fixation disc disappeared and a target concurrently appeared either to the left or the right at 11° of visual angle. The fore-period delay between reward cue and target onset allowed effects of reward processing and any movement effect on pupil dilation sufficient time to uncouple.^45^ Rewards earned were displayed in the centre of the target after completion of each trial. The total amount of reward received on each trial was performance driven dependent on saccadic reaction time (RT).

#### ‘No-go’ trials

No-go trials were indicated by a ‘×’ symbol at the start of the trial. In this condition, the target always remained at the centre of the screen and participants had to maintain fixation. Fixation was held for the same three variable fore-periods of either 2000, 2100 or 2200 ms as in Go trials. Participants were instructed that the proportion of reward earned depended on their ability to stare centrally at the target. Earnings were displayed in the same way as in ‘go’ trials.

The two types of trial occurred in equal frequency in a randomised order. Trials were separated by a 2500 ms interval and divided into blocks of 36 trials. Young participants completed eight blocks divided into two sessions separated by a 10-min break. Elderly controls and Parkinson’s disease patients completed six blocks divided into two equal sessions separated by a 10-minute break. Each block lasted approximately 4.5 min.

Parkinson’s disease patients were tested ON and OFF their normal dopaminergic medication. In the OFF condition they were asked not to take any of their dopaminergic medication on the morning of the study and were therefore OFF medication following an overnight fast of approximately 14 h (Table 1).

### Reward criteria

Participants received a proportion of the maximum reward on offer and was based on performance. In the ‘go’ condition, the amount of reward earned varied with RT and dynamically adjusted using an adaptive exponential fall-off based on average RT of the preceding twenty trials. For further details see Muhammed *et al*.^16^ methods. Unbeknown to the participants, the average earnings in the last 20 trials of the ‘go’ condition determined the reward obtained in ‘no-go’ trials. This ensured that the same overall amounts of money were received in the two conditions over the course of the experiment. Trials where fixation was not maintained throughout the ‘no-go’ condition were excluded from analysis, this accounted for less than 5% of trials.

### Eye tracking recording

Pupil dilation was calculated as a proportional change from average baseline pupil size measured in Eyelink units. Mean pupillary size from 1400 to 2400 ms after the auditory cue was used as the time period of interest as per previous studies.^15^^,^^16^^,^^46^ It was selected to allow sufficient time for pupillary changes to uncouple between different rewards on offer while also minimizing the effects of subsequent saccades that are made when the target appears. The duration of 1000 ms is of long enough duration to capture meaningful differences in the pupil while minimizing noise related to other elements of the trial. An individual's ‘pupillary reward sensitivity’ was defined as the difference in mean proportional pupil change between the maximal 50p reward and 0p reward conditions, over the epoch of interest. A larger difference indicated greater reward sensitivity.

### Eye tracker data statistical analysis

Analysis was performed using repeated measures analysis of variance (ANOVA). To account for any non-sphericity in the data, where appropriate, statistics are reported with Greenhouse-Geisser correction. Due to clear assumptions and results from previous studies, significant main effects and interactions were decomposed with planned contrasts. Significance was taken as *P*-values of less than 0.05. Correlations with questionnaires were performed using Spearman rank non-parametric testing and Pearson correlations were used for parametric behavioural outcome comparisons. To correct for multiple comparisons when calculating differences in pupil traces over time, permutation testing was performed. Epochs were averaged to obtain an average pupil size for each condition for each participant over time. These averages were then permuted randomly for each participant, to create a new dataset with rearranged condition labels. The *t*-statistics for the contrast of interest for each random permutation was calculated at each time point along the trace. The maximum of this *t*-statistic was computed for 5000 permutations creating a null distribution of the maximum *t*-statistics. *P*-values were then generated by comparing the *t*-statistics of the original (unpermuted) data at each time-point with the null distribution. Those timepoints with probability less than 0.05 are displayed as continuous solid horizontal bars. Statistics were completed using Matlab and SPSS.

### Data availability

The data that support the findings of this study are available from the corresponding author upon request.

## Results

### Pupillary response to rewards in ‘Go’ and ‘No-go’ trials

**Figure 2:**
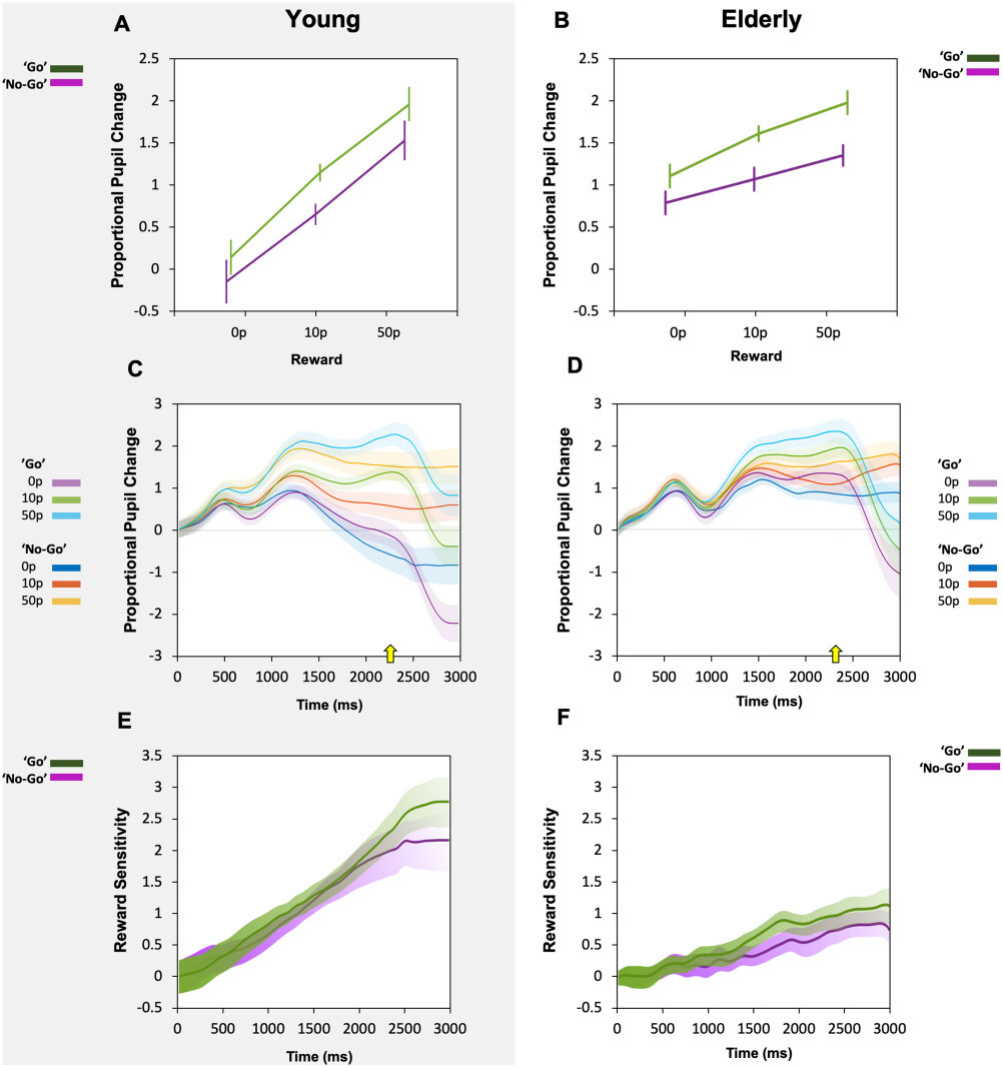
**Pupil responses in young (A, C, E) and elderly (B, D, F) participants across ‘go’ and ‘no-go’ trials.** (A) Pupil proportional change to reward taken as an average across a 1000 ms epoch from 1400 ms to 2400 ms in young participants. Increased pupil response to reward was present as reward increased but no difference was seen between ‘go’ trials in green and ‘no-go’ trials in violet. (**B**) Pupil proportional change to reward in elderly participants. Increased pupil response to reward was present as reward level increased but like in the young, no difference was seen between ‘go’ trials (green line) and ‘no-go’ trials (violet line). (**C**) Average proportional pupil changes over the course of the trials in young participants, broken down into responses to ‘go’ and ‘no-go’ cues. Cue onset was at time 0 ms. Average onset of saccades indicated by yellow arrow (2296 ms). (**D**) Average proportional pupil changes over the course of the trials in the elderly. Average onset of saccades indicated by yellow arrow (2347 ms). (**E**) Average reward sensitivity over time in young participants, taken as the difference in the proportional pupil change between the 50p reward condition and the 0p reward. Broken into reward sensitivity for ‘go’ trials—green, versus ‘no-go’ trials—violet. No significant difference in reward sensitivity was present at any time point across the length of the entire trial. (**F**) Average reward sensitivity over time in the elderly, calculated as the difference in the proportional pupil change between the 50p reward condition and the 0p reward for each time point. Reward sensitivity for ‘go’ trials plotted in green and ‘no-go’ trials in violet. No significant difference in pupil reward sensitivity was present.

Using the 1400 ms to 2400 ms epoch of interest, the change of pupil size as a proportion of baseline when anticipating a reward in ‘go’ and ‘no go’ trials was examined. In young healthy participants, greater rewards on offer led to larger pupil modulation [main effect of reward (*F*(2, 44) = 19.15, *P *<* *0.0001)]. Crucially, this pupillary response to reward was present *both* when an action had to be made in the ‘go’ condition, and also when no action was needed in the ‘no go’ condition. There were no significant effects of ‘go’ or ‘no-go’ trial type (*F*(1, 22) = 3.7, *P *=* *0.07) and no interaction (*F*(2, 44) = 0.158, *P *=* *0.85) (Fig. 2A). The pupillary response to reward was present both in the ‘go’ and the ‘no-go’ condition in the elderly group as well [main effect of reward (*F*(2, 44) = 14.4, *P *<* *0.0001)]. Like in the young, there was no significant main effect of ‘go’ or ‘no-go’ trials (*F*(1, 22) = 3.2, *P *=* *0.09) and no interaction (*F*(2, 44) = 0.19, *P *=* *0.83), but a suggestion that the ‘go’ trials lead to larger pupil dilation overall was still evident although not significant (Fig. 2B).

In the young, pupil change over time demonstrated divergence of pupil response for the three reward levels (Fig. 2C). The pupil responses in the ‘go’ and the ‘no-go’ trials had different patterns of dilation and constriction responses for reward. In the ‘go’ arm, a tri-phasic dilation and constriction pattern was seen, with slightly greater peaks in pupil dilation overtime, whereas the ‘no-go’ trials had a more bi-phasic response, and overall flatter pupillary changes over time. Target appearance was from 2000 to 2200 ms and RT was approximately 200 ms, so saccadic onset ranged from approximately 2200 ms to 2400 ms (average saccade onset is indicated by yellow arrow Fig. 2C and D). The time a saccade was initiated coincides with the different patterns of pupil response between the ‘go’ and ‘no-go’ conditions and likely accounts for the variation in dilation and constriction demonstrated. However, when comparisons were made over the 1000 ms average epoch of interest (1400 ms to 2400 ms; Fig. 2A), no overall difference was found statistically. To determine if any association between pupil dilation and saccades were present, a correlation analysis was performed for each reward magnitude between saccadic velocity and subsequent pupil dilation. When accounting for multiple comparisons no significant correlation was found. RT did not correlate with any changes in pupil size regardless of reward on offer in all groups.

Pupil change over time for the different conditions and reward levels in elderly participants, again revealed slightly different patterns of pupil dilation for reward when an action needed to be made versus when none was needed (Fig. 2D). On average, divergence of pupil response for the different reward levels was slightly later and appeared to differ the greatest between the ‘go’ and ‘no-go’ condition around the onset of saccades in the ‘go’ trials (Fig. 2D, yellow arrow). Like the young participants, despite the differences in dilation pattern no statistical significance for pupil responses to reward was reached between the ‘go’ condition compared to the ‘no-go’ condition (Fig. 2B).

### Reward sensitivity

In the young group, it seemed there may be a larger overall pupil response to each reward level in the ‘go’ condition compared to the ‘no go’. However, when comparing reward sensitivity (50p pupil response minus 0p pupil response) in the ‘no-go’ trials versus the ‘go’ trials, across the entire trial period, there were no significance differences detected (Fig. 2E). Comparisons were made using multiple permutation tests at each millisecond time point. Like in the young, pupil reward sensitivity in ‘no go’ versus ‘go’ conditions across the whole trial period was not significant in elderly controls either (Fig. 2F).

### Pupillary comparisons in young versus elderly

**Figure 3:**
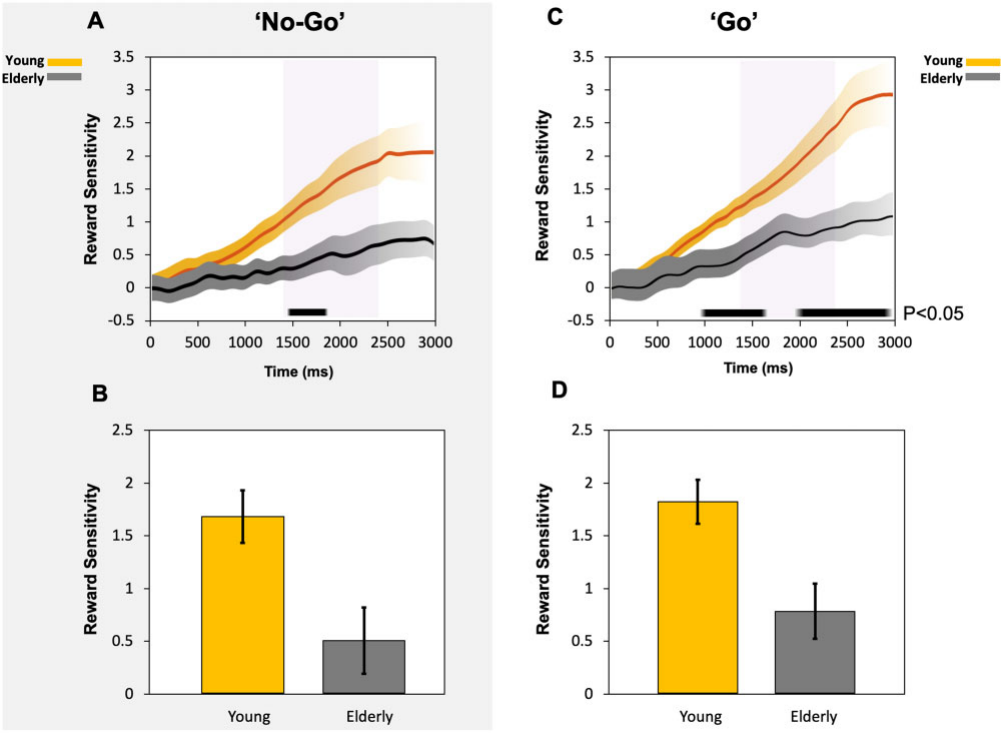
**Reward sensitivity in ‘no-go’ (A, B) and ‘go’ (C, D) trials compared between young and elderly groups.** (**A**) Average reward sensitivity over time in ‘no-go’ trials, taken as the difference in the proportional pupil change between the 50p reward condition and the 0p reward. Broken into reward sensitivity for young (yellow) and elderly participants (grey). Black bar denotes significant difference between the two groups over a short ∼350 ms period. (**B**) Pupil reward sensitivity (50p-0p reward) taken as an average across a 1000 ms time epoch from 1400 ms to 2400 ms in the ‘no-go’ trials (Purple background shading in A). Young participants had significantly larger pupil response to reward over the time epoch of interest compared to elderly participants. (**C**) Average reward sensitivity over time in ‘go’ trials, taken as the difference in the proportional pupil change between the 50p reward condition and the 0p reward. Reward sensitivity in young participants (yellow) and elderly (grey). Black bar denotes significant difference between the two groups over a large period of the trial starting from ∼1000 ms post reward cue onset to the end of the trial. (**D**) Pupil reward sensitivity (50p-0p reward) taken as an average across a 1000 ms period from 1400 ms to 2400 ms in the ‘go’ trials (Purple background shading in C). Young participants had significantly larger pupil response to reward compared to elderly participants.

As above, pupil reward sensitivity was calculated as the pupil response to the 50p reward minus the 0p response over time. Pupil reward sensitivity in young controls compared to elderly controls in the ‘no-go’ trials using multiple permutation tests showed a small ∼350 ms time period of differences between the two groups (*P* < 0.05) (Fig. 3A; delineated by black bar). The difference between the two groups became much more prominent when the ‘go’ trials were examined over time (Fig. 3C; black bar). A large duration of difference from around ∼1000 ms to the end of the trial was evident using multiple permutation testing, there was only a short period where statistical significance was not reached at a two-tailed level (*P* < 0.05).

Using ANOVA to compare the average reward sensitivity of the young controls versus elderly controls in the ‘no-go’ trials over the 1400 ms to 2400 ms epoch of interest, there was a borderline significant difference (F(1, 40) = 4.2, *P *=* *0.048). Young controls had a higher reward sensitivity compared to elderly controls, in the ‘no go’ condition (Fig. 3B). This difference was more so when comparing the two groups pupil reward sensitivity in the ‘go’ trials (F(1, 40) = 4.6, *P *=* *0.038; Fig. 3D). No correlation between pupil reward sensitivity and saccadic velocity was present in young or elderly participants.

### Parkinson’s disease ON and OFF

#### Pupillary response to rewards in ‘go’ and ‘no-go’ trials

**Figure 4:**
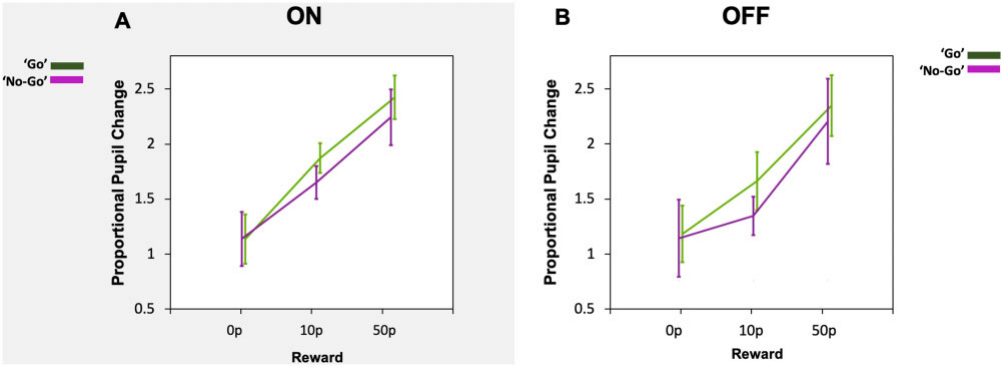
**Parkinson’s disease pupil response to reward in ‘go’ and ‘no-go’ trials in the ON and OFF state.** (**A**) When ON dopamine, pupil proportional change to reward taken as an average across a 1000 ms period of time from 1400 ms to 2400 ms. Increased pupil response to reward was present as reward magnitude increased but no difference was seen between ‘go’ trials (green) and ‘no-go’ trials (violet). (**B**) When OFF dopamine, pupil proportional change also increased as reward increased, with no difference in ‘go’ trials (green) and ‘no-go’ trials (violet).

Using average pupil changes over the 1000 ms time period of interest (1400–2400 ms), comparison of pupil response to rewards in the ‘go’ versus the ‘no-go’ condition was made in Parkinson’s disease patients. Similar findings were present in both dopaminergic states. When ON (Fig. 4A) or OFF (Fig. 4B) dopamine, a significant main effect of reward was present (*F*(2, 42) = 8.4, *P *=* *0.001; *F*(2, 42) = 3.6, *P *=* *0.037, respectively), with increasing pupil dilation for increasing reward magnitude. There were no main effects of trial condition [‘go’ and ‘no-go’, *F*(1, 21) = 0.68, *P *=* *0.42 when ON and *F*(1, 21) = 0.38, *P *=* *0.54 when OFF], nor any interaction (*F*(2, 42) = 0.31, *P *=* *0.73 ON and *F*(2, 44) = 0.51, *P *=* *0.61 when OFF). Finally, no correlations between pupil dilation and saccadic velocity or RT were present in Parkinson’s disease, both when ON or when OFF dopaminergic medication.

#### Dopamine and pupil response of ‘go’ versus ‘no-go’ trials

When comparing ON and OFF, ‘go’ and ‘no-go’ effects on pupil response using a repeated measures ANOVA, only a significant main effect of reward was present (*F*(2, 42) = 6.7, *P *=* *0.003). No other main effects or interactions were found. Planned contrasts demonstrated a significant difference between all reward levels (*P* < 0.02) except between the 0p and 10p reward (*P* = 0.073). Therefore, dopamine did not significantly alter pupil response to rewards when a saccade was made (‘go’ trials) versus when it was not (‘no-go’ trials), but increasing reward levels modulated pupillary response, irrespective of dopaminergic state.

**Figure 5:**
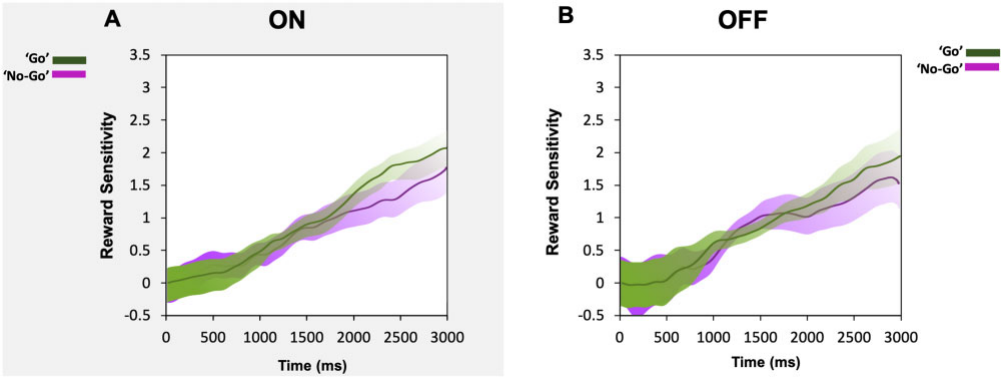
**Parkinson’s disease pupil reward sensitivity during ON (A) and OFF (B) dopamine states.** (**A**) Average reward sensitivity over time in Parkinson’s disease when ON in ‘go’ trials (green) compared to ‘no-go’ trials (violet) taken as the difference in the proportional pupil change between the 50p reward condition and the 0p reward. Using permutation testing at each time point, no significant difference was demonstrated between the two conditions over the course of the trial. (**B**) Average reward sensitivity over time in Parkinson’s disease when OFF in ‘go’ trials (green) compared to ‘no-go’ trials (violet). No significant difference was present between the two conditions either.

This lack of difference was also true for pupil reward sensitivity (50p pupil response minus 0p pupil response) across the course of the trial regardless of whether a ‘go’ or a ‘no-go’ trial was performed or if ON or OFF dopamine. This shows that reward sensitivity in pupil response over time in either the ON state or OFF state is not contingent on making an action (Fig. 5).

#### Pupil reward sensitivity and a link to motivation

**Figure 6:**
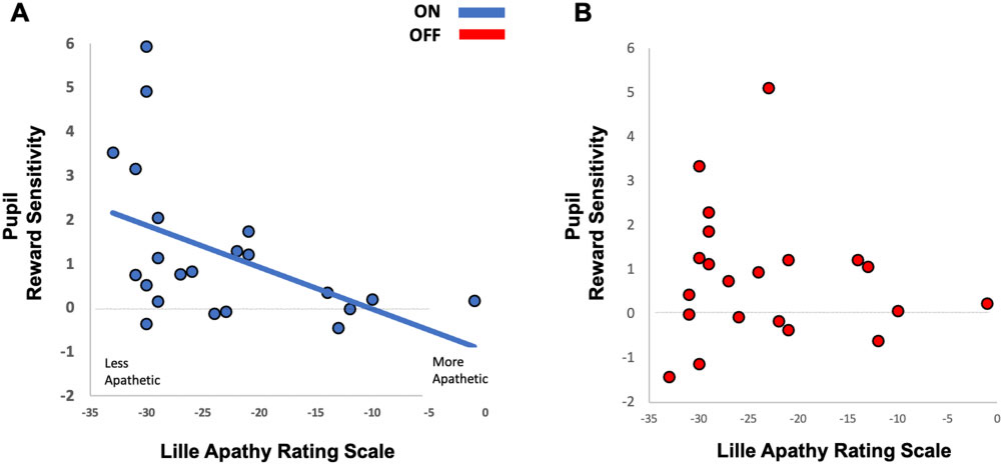
**Correlation with clinical apathy questionnaire scores (Lille Apathy Rating Scale) and pupil reward sensitivity when a saccade was made to obtain a reward in Parkinson’s disease patients ON and OFF dopamine.** (**A**) A significant correlation between average pupil reward sensitivity in Parkinson’s disease patients ON (blue) and their clinical interview LARS score was present only in the ‘go’ trials. More apathetic patients exhibit less pupillary reward sensitivity compared to more motivated patient. Spearman correlation, rs = −0.472, P < 0.03. Mean apathetic patients reward sensitivity in ‘go’ trials ON dopamine 0.56 (SD 0.76). Non-Apathetic patients mean 1.65 (SD 1.98). No correlation was demonstrated in ‘no-go’ trials when ON or OFF dopamine. (**B**) The correlation between average pupil reward sensitivity in Parkinson’s disease during ‘go’ trials and LARS score was abolished when OFF dopamine (red). Spearman correlation, rs = −0.091, P = 0.686. Mean apathetic patients reward sensitivity in ‘go’ trials OFF dopamine 0.33 (SD 0.7). Non-Apathetic patients mean 1.68 (SD 2.7).

Comparison of reward sensitivity in the ‘go’ and the ‘no-go’ condition to clinical apathy levels in Parkinson’s disease (indexed by the LARS) produced some noteworthy results. The total LARS score correlated significantly with pupil reward sensitivity in the ON state, but only in the ‘go’ condition, *r_s_* = −0.472, *P* < 0.03 (Fig. 6A, Blue line). Further, the action initiation sub score of the LARS also only correlated with pupil reward sensitivity in the ‘go’ condition, *r_s_* = −0.476, *P* = 0.025. In the ‘no-go’ condition, where no initiation of action was required, there was no significant correlation with either total LARS or the action initiation sub scores (*r_s_* = −0.116, *P* = 0.608 and *r_s_* = −0.029, *P* = 0.897, respectively). When OFF dopamine these effects were abolished, LARS and ‘go’ pupil reward sensitivity no longer showed a significant correlation (*r_s_* = −0.091, *P* = 0.686) (Fig. 6B). Thus, pupillary reward sensitivity was significantly blunted with increasing apathy only on ‘go’ trials in the ON state. To factor in the effect of age on reward sensitivity a multiple regression analysis was performed. A multiple regression analysis with pupil reward sensitivity as the dependent variable and LARS apathy score and age as the regressor variables found age not to be a significant predictor in the ‘go’ or ‘no-go’ condition either ON or OFF dopamine. There was no significant correlation with either the ‘go’ or the ‘no-go’ pupil response in Parkinson’s disease ON or OFF with other clinical assessments including depression or cognitive function as measured by the BDI-II and the MoCA.

#### Pupillary response to rewards in Parkinson’s disease versus elderly controls

**Figure 7:**
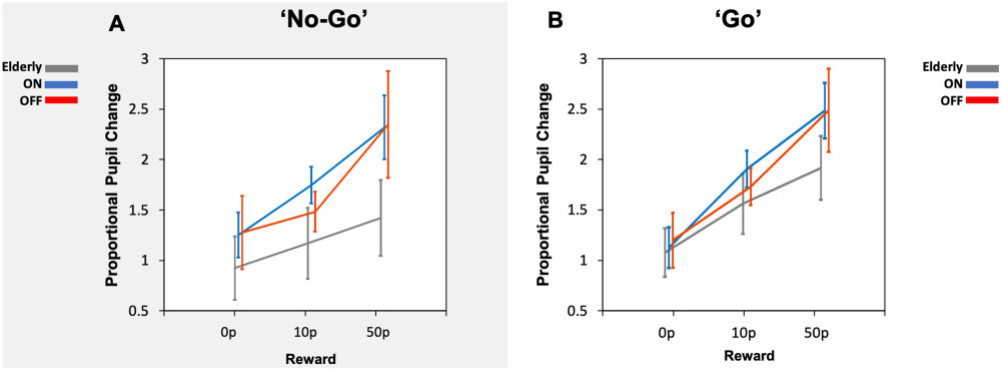
**Parkinson’s disease and elderly pupil response to reward in the ON and OFF state during ‘no-go’ (A) and ‘go’ (B) trials.** (**A**) No differences in the average pupil change between ON, OFF or elderly controls in ‘no-go’ trials were present either, however all groups demonstrated increases in pupil size for larger reward levels. (**B**) Average pupillary response over the time epoch of interest (1400–2400 ms) in all groups displayed an increase in pupil size for increasing reward level in ‘go’ trials. There were no differences in the average pupil change between ON (blue), OFF (red) or elderly controls (grey).

Finally, pupil comparisons were made with Parkinson’s disease patients ON and OFF dopamine, and elderly controls. Parkinson’s disease ON versus elderly controls and Parkinson’s disease OFF versus elderly controls comparisons were made separately. Comparing mean pupil change across the time period of interest (1400 ms to 2400 ms) in the ‘go’ condition between elderly controls and Parkinson’s disease patients ON showed only a main effect of reward (*F*(2, 76) = 7.0, *P *=* *0.002, Fig. 7B). There were no main effects of group or interactions. Similarly, the ‘no-go’ condition comparison between Parkinson’s disease ON and elderly controls also demonstrated a main effect of reward (*F*(2, 76) = 16.1, *P *<* *0.00001) but no other significant differences (Fig. 7A). Pupil comparisons in Parkinson’s disease patients when OFF dopamine and in the elderly also revealed a main effect of reward in both the ‘no-go’ (Fig. 7A) and the ‘go’ conditions (Fig. 7B) (*F*(2, 76) = 8.6, *P *<* *0.001) and (*F*(2, 76) = 3.9, *P *=* *0.025), respectively. There were no other main effects or interactions, suggesting that only the modulatory effect of reward was present in all groups regardless of whether a saccade was initiated or not.

#### Other oculomotor proprieties in young, elderly and Parkinson’s disease

For increasing magnitudes of reward on offer, saccadic velocity increased while accuracy did not worsen. This was true in young, elderly and Parkinson’s disease patients. Invigoration of saccades without worsening of accuracy appears to break speed accuracy tradeoffs and provides further evidence that motivation by reward can improve both speed and accuracy.^21^ Full results from saccadic velocity, amplitude variability and RT for all groups are presented in the Supplementary Materials.

## Discussion

The findings presented here demonstrate that pupil responses to incentives are *not* contingent on action: They are present *both* when initiation of an action is required to obtain a reward (‘go’ trials) and when no action is necessary (‘no-go’ trials). This was true in young and elderly healthy participants (Fig. 2A and B) as well as patients with Parkinson’s disease (Fig. 4). No robust associations between anticipatory pupil response and subsequent saccadic velocity and RT to obtain the reward were found. This suggests that anticipatory pupillary response to reward reflects evaluation of cued incentives rather than motor preparation. The findings that pupil reward sensitivity reduced with age (Fig. 3) replicated results from a previous pupillometry study of motivation,^16^ but show that this aging effect occurs regardless of whether actions have to be made.

### Reward sensitivity and apathy in Parkinson’s disease

The results demonstrated a significant association between pupillary reward sensitivity and clinical apathy in Parkinson’s disease, again replicating findings in a previous study.^16^ However, this effect was obtained only on ‘go’ trials and while ON dopamine. Thus, more apathetic patients showed less pupillary reward sensitivity when they had to make a speeded saccade in order to obtain a reward, but only when ON dopaminergic medication (Fig. 6A). This finding suggests that apathy in Parkinson’s disease is indeed linked to reduced reward sensitivity when actions have to be initiated to rewarding goals, and this effect is modulated by being ON dopaminergic medication. OFF medication, there was no such relationship (Fig. 6B), and similarly no association with apathy and pupillary reward sensitivity in the ‘no-go’ conditions, either ON or OFF dopaminergic drugs. Differences in variance between apathetic patients and non-apathetic patients could be contributing to these results. However, in Parkinson’s disease OFF ‘go’ trials in non-apathetic individuals, variance was greater than when ON and no correlation was demonstrated. Therefore, this alone cannot account for the significant result in the Parkinson’s disease ON ‘go’ condition.

The links between initiation of actions and reward sensitivity is of particular interest in the context of apathy in Parkinson’s disease. Patients with severe behavioural apathy, sometimes termed an auto-activation deficit,^33^^,^^47^ have a significant reduction in goal directed behaviours,^6^ often describing a lack of drive to initiate and carry out everyday tasks.^26^ Pupil dilation for reward cues in this study were associated with apathy level only when an action (saccadic eye movement) had to be made to obtain the reward. This finding is reminiscent of work in a rodent study where phasic dopamine spikes in the ventral striatum (NAcc specifically) occurred only when motor action was also needed to receive a reward.^35^ The result suggests an interaction between motivation and motor control^48^ with the NAcc potentially being a crucial interface between motivation and motoric action.^36^^,^^37^

In apathetic individuals, this interface may be dysfunctional, with the gain relating evaluation of rewards (reward sensitivity) to initiation of action in order to obtain them significantly reduced.^17^ In our study, when Parkinson’s disease patients were (relatively) more dopamine depleted in the OFF state, the association between reward sensitivity and apathy was abolished. One might speculate that this is due to a reduction of available dopamine within the striatum, impairing the link between limbic circuits involved in motivation and motor pathways. Hence, the relationship between the valuation of rewards and initiation of action might be central to motivational processes.^27^^,^^28^^,^^34^ In this context, it might be relevant that degeneration of the dopaminergic VTA-NAcc pathway in MPTP lesioned monkeys has also been linked with apathy ^40^ while injections of bicuculline, a GABA antagonist, into the ventral striatum of monkeys also leads to a hypoactive state.^49^

Nevertheless, it would be important not to speculate too much. It might simply be that the relationship between apathy and pupil response to reward is strongest when Parkinson’s disease patients have to make actions and in the ON condition, and in the other conditions the effect is not so reliable. Furthermore, it is noteworthy that although dopamine modulated the association between reward sensitivity and apathy in Parkinson’s disease, there were no differences in overall pupil reward sensitivity when ON or OFF dopamine, irrespective of whether an action was initiated or not (Fig. 7). This finding does not replicate previous work by Muhammed *et al*.^16^ which demonstrates increased overall reward sensitivity when ON dopamine in Parkinson’s disease. The lack of modulation cannot be accounted for by disease duration or total dopamine dose between the two studies. The average disease duration of Parkinson’s disease patients in both studies was equal at 5 years. Patients in this study on average had higher levodopa equivalence doses, however the type of dopaminergic medication differed. In this study cohort, 59% of the patients were taking a dopamine agonist either alone or as part of their treatment regimen versus 70% in the original study. Dopamine agonists may boost reward sensitivity to a greater degree than levodopa alone^46^ and this may account for some of the differences observed here. The pupillary links with apathy in this study are an interesting finding. However, due to smaller numbers of patients who were classified as clinically apathetic, further follow-up investigations with a larger sample size is merited in order to perform more reliable sub-group analysis.

In addition, the disease phenotype of patients may also be a factor contributing to the lack of dopamine modulation. Parkinson’s disease is a heterogeneous condition with some patients more dopamine responsive than others. For example, those with akinetic-rigid variant Parkinson’s disease are less likely to show dopamine responsiveness than individuals with tremor-dominant Parkinson’s disease, and are also worse affected by apathy.^50^ Also, in clinical practice, motivational dysfunction is not always responsive to dopamine, leading to suggestions of two types of a motivation syndromes. The first is dopamine-sensitive apathy which, for example, has been observed post deep brain stimulation insertion following dopamine withdrawal and can be reversed after reintroduction of medication.^51^^,^^52^ The second is dopamine-resistant apathy, which may be related to the progression of Parkinson’s disease and atrophy of the NAcc and caudate nucleus.^53^

### Oculomotor properties and trial categories

Overall, dopamine also did not change the pupil response to rewards when a saccade was made (‘go’) versus not made (‘no-go’) in Parkinson’s disease, with modulation by reward observed *irrespective* of movement. This is an important finding as it demonstrates no link between action-dependent preparatory motivation (indexed by pupillary dilation to reward) and dopamine (Fig. 5). Irrespective of drug state, pupil responses in Parkinson’s disease, both when initiation of an action was needed to obtain a reward and when no action was necessary, increased with larger reward magnitudes (Fig. 4). Although dopamine did not affect pupillary reward sensitivity, nor its association with motor action, it did modulate *saccadic* reward sensitivity. Parkinson’s disease patients ON dopamine showed a significant increase in peak velocity for rewards of larger magnitude and this difference was present between each reward level. When in the OFF state, the extent to which peak velocity increased with reward was reduced although a main effect of reward was still present (see Supplementary Fig. 4A).

Saccades are known to be invigorated by rewards^16^^,^^54^^,^^55^ and there is also evidence for motor preparation signals in various saccadic tasks, particularly when movements are primed.^56–59^ Recordings from oculomotor brain regions such as the frontal eye fields demonstrate ramping up of activity prior to saccade onset.^60^ It would be important to establish if changes in pupil response attributed to reward cues are indeed due to reward evaluation rather than preparation for motor action for upcoming saccades. In our study, pupil modulation for prospective rewards prior to making a saccade compared to when no movement was required could be assessed. No significant differences were observed between the trial types, with an equal rise in reward sensitivity on increasing magnitude of incentive offered irrespective of an action being executed. There were also no associations between pupil dilation and saccadic velocity or RT in Parkinson’s disease regardless of dopamine state. These results strengthen the conclusion that pupil response to available reward prior to the onset of an eye movement is not linked to saccadic velocity or action onset latency in this task. Other oculomotor properties including saccadic velocity, RT and accuracy were also not correlated with apathy, but rewards did invigorate saccadic velocity *without* impairing accuracy (see Supplementary Fig. 4A and B). This finding breaks classical observations of speed accuracy trade-offs, reproducing recent results in the literature.^16^^,^^20^^,^^21^^,^^54^^,^^55^^,^^61^

Unlike a traditional ‘no-go’ condition where response inhibition is required, the ‘no-go’ trials in this study were intentionally designed to minimize motor preparation changes in the pupil while only varying the reward obtained to match the ‘go’ conditions. We acknowledge however that although no motor preparation was required in the ‘no-go’ trials, there will be a degree of control needed to maintain fixation which could influence the pupil size and could account for similarities in the results between the two conditions. Feedback from participants however indicated that the ‘no-go’ trials were not considered to be demanding. Indeed, they reported that even though they needed to remain fixated on the target during these trials, they considered them significantly less demanding in comparison to the ‘go’ condition as no motor action was needed. Fixation was also only maintained for a maximum duration of 2200 ms and the fixation circle was large minimizing the difficulty to maintain fixation.

### Reward sensitivity in young and elderly

Reward sensitivity in healthy people—both young and old—was not dependent on action initiation. However, there appeared to be a stronger response to reward in young versus elderly participants, with larger pupillary reward sensitivity differences over time when a saccade was initiated (‘go’ trials) (Fig. 3C and D) compared to when no movement was made (‘no-go’ trials) (Fig. 3A and B). The reduction in reward sensitivity with age may be related to differences in dopaminergic neuromodulation. Findings from functional MRI (fMRI) studies demonstrate lower ventral striatal activation to immediate reward in the elderly and reduced activity in the dorsal striatum in comparison to younger adults.^62^ The dorsal striatum is associated with motor output with a gradient of function within the striatum which transitions from the intention to act to the movement itself, in a ventral to dorsal direction.^63^ These striatal differences may explain the reduction in reward sensitivity in the elderly observed in our study, especially when an action was required to be made. Potential future work could include the use of fMRI in conjunction with oculomotor tasks to help identify pertinent brain regions.

## Conclusion

In conclusion, the findings from this study adds to our understanding of reward sensitivity and interpretation of the mechanisms underlying apathy. From a movement perspective, it appears pupil response to rewards do not arise as a consequence of motor preparation signals linked to performing a saccade. Whether an action is required or not, reward appears to modulate pupil reward sensitivity in the young, elderly and patients with Parkinson’s disease both ON and OFF dopamine. Although direct conclusions about dopamine striatal signalling cannot be made from this study, the association with pupil reward sensitivity and motivation when an action is required aligns with findings in the animal literature suggesting phasic dopamine spikes in the ventral striatum occur only when motor action is also needed to receive a reward.^35^ The findings provide some evidence to suggest that apathy is associated with a reduction in reward sensitivity in Parkinson’s disease but only when paired with the initiation of a goal directed action, and this link appears to be strongest and most evident when patients are ON dopaminergic medication.

## Supplementary material

Supplementary material is available at *Brain Communications* online.

## Funding

K.M. was supported by a Wellcome Trust Clinical Research Training Fellowship and a National Institute for Health Research Clinical Lectureship; M.H. is supported by a Wellcome Trust Principal Research Fellowship and by the National Institute for Health Research Oxford Biomedical Research Centre.

## Competing interests

The authors report no competing interests.

## Supplementary Material

fcab022_Supplementary_Data

## Abbreviations

ANOVA =: analysis of variance
BDI-II =: Beck Depression Inventory-II
fMRI =: functional MRI
LARS =: Lille Apathy Rating Scale
MoCA =: Montreal Cognitive Assessment
MPTP =: 1-methyl-4-phenyl-1,2,3,6-tetrahydropyridine
NAcc =: Nucleus accumbens
RT =: Reaction time
UPDRS =: Movement Disorder Society Unified Parkinson’s disease rating scale
VTA =: Ventral tegmental area
