# Supplementary material for: Reward sensitivity and action in Parkinson’s disease patients with and without apathy
Source: Brain Commun. 2021 Mar 10;3(2):fcab022. doi: 10.1093/braincomms/fcab022 (PMC8024004; doi:10.1093/braincomms/fcab022)
Supplement: fcab022_Supplementary_Data [file fcab022_supplementary_data.pdf]

Reward sensitivity and action in Parkinson’s disease patients with and without apathy

Supplementary Materials

Young Control Participants:

Saccadic peak velocity

The saccadic properties in the 'Go' arm were analyzed. Saccadic peak velocity showed increases in speed for larger rewards compared to no rewards with a main effect of reward, ($F(2, 44) = 25.8$, $P < 0.0001$), and significant differences in velocities between the 0p versus 10p reward and the 0p versus 50p reward ($p<0.001$), but no differences between the 10p versus 50p condition were present ($p=0.21$). (**Fig. S1A**).

Saccadic accuracy

Accuracy, as assessed by mean variability in saccadic amplitude, did not demonstrate a within subjects main effect of reward ($F(2, 44) = 1.36$, $P = 0.276$), however planned within subject contrasts demonstrated a significant linear improvement in accuracy for the higher 50p reward compared to no reward, ($F(1, 2) = 8.13$, $P = 0.009$), significant for the 0p versus 50p comparison. Therefore, accuracy did not worsen despite saccadic velocity for bigger rewards being faster, breaking speed accuracy tradeoffs. (**Fig. S1B**).

Saccadic reaction time

There were no significant effects of reaction time in this task, ($F(2, 44) = 1.6$, $P = 0.2$). (**Fig. S1C**).

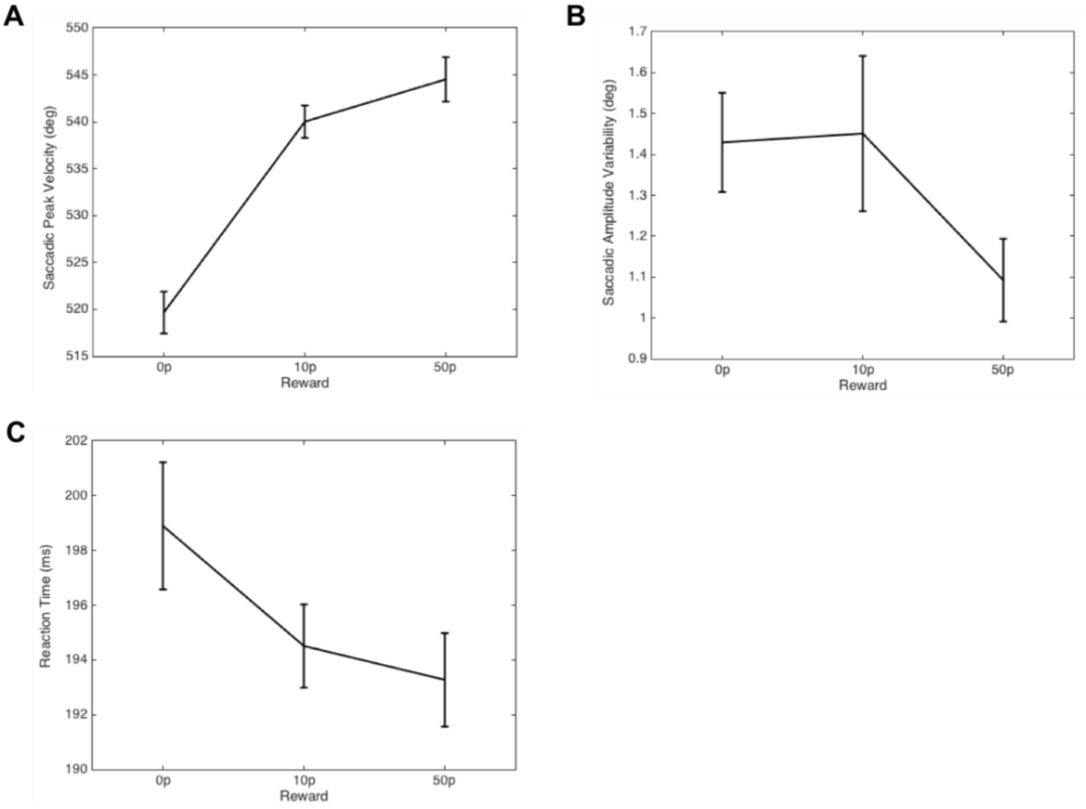

Figure S1 A-C. Oculomotor responses from young healthy participants in the 'go' arm of the experiment.

A. *Saccadic peak velocity measured in degrees per second increased for larger magnitudes of reward on offer.*

B. *Accuracy as measured by saccadic variability as a function of incentive on offer. A significant reduction in saccadic variability for the largest reward level compared to no reward was present.*

C. *Reaction time for increasing reward levels did not reach statistical significance.*

Elderly Controls Participants:

Saccadic peak velocity

Saccadic peak velocity in the elderly participants increased only for the largest 50p reward level, driving a significant main effect of reward (F(2, 34) = 6.8, *P* = 0.003). There was a significant difference in peak velocity between the 10p versus 50p reward and the 0p and

50p reward ($p<0.02$), but no differences between the 0p versus 10p condition ($p=0.83$). (**Fig. S2A**).

Saccadic accuracy

Variability in saccadic amplitude did not demonstrate a main effect of reward in the elderly ($F(2, 34) = 0.879$, $P = 425$). Although there was no improvement in accuracy with increasing reward, there was also no reduction in accuracy despite an increase in velocity being present with the largest reward, which also defies speed accuracy trade off predictions and is consistent with the previous findings in the young. (**Fig. S2B**)

Saccadic reaction time

Much like the young participants, there were no significant effects of reaction time in the task in the elderly controls, ($F(2, 34) = 1.8$, $P = 0.83$). (**Fig. S2C**)

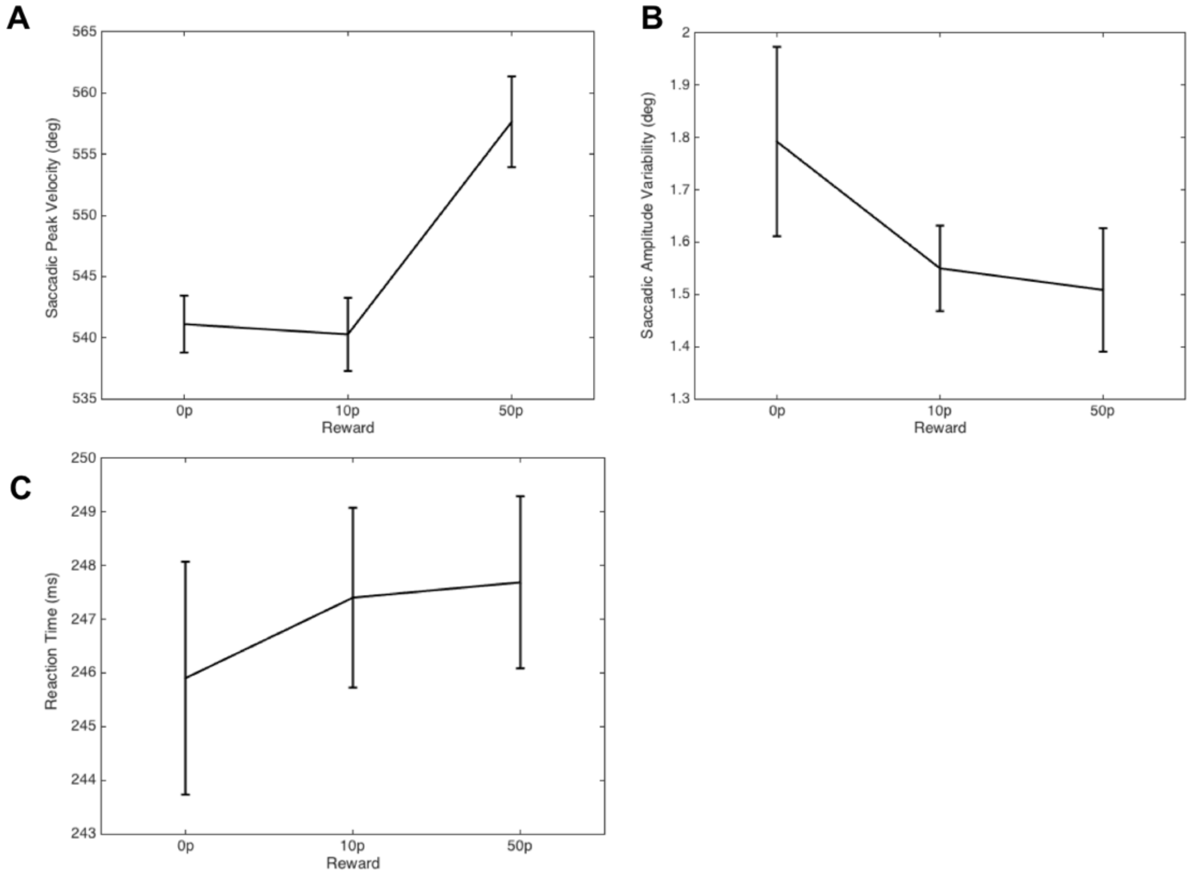

Figure S2A-C. Oculomotor responses from elderly healthy participants.

- **A.** *Saccadic peak velocity as a function of incentive. Saccadic velocity increased for the largest 50p level of reward on offer compared to the 0p and 10p level.*
- **B.** *Saccadic accuracy as assessed by variability of saccade amplitude. There was no significant difference in accuracy as reward levels increased in elderly participants.*
- **C.** *Reaction time for increasing reward levels did not differ between reward levels in the elderly group.*

Young versus Elderly Controls:

Saccadic peak velocity

Comparing peak velocities in the 'go' arm of the experiment between the young and the elderly groups demonstrated a main effect of reward ($F(2, 78) = 21.3$, $P < 0.001$), with faster speeds between each reward level ($p<0.01$). Interestingly, there was also a reward by group interaction ($F(2, 78) = 5.8$, $P < 0.01$). Deconstructing this interaction revealed that the

young controls showed the biggest difference in velocity between the 0p versus 10p reward and the 0p versus 50p reward ($p<0.001$), with no difference between the 10p versus 50p condition. Whereas the elderly group demonstrated the greatest difference between the 0p versus 50p and the 10p versus 50p but not the 0p versus 10p condition. This suggests that there may be a shift in the importance attributed to various reward sizes with aging, perhaps with more elderly individuals needing greater amounts of reward to speed up saccades and the young attributing the same value to all rewards greater than 0p. (**Fig. S3A**).

Saccadic accuracy

Variability in saccadic amplitude showed no main effect of reward ($F(2,78) = 1.7$, $P = 0.199$), but there was a within subject linear contrast effect of reward ($F(1,39) =$, $P = 0.04$) suggesting that the difference between the largest 50p reward and 0p level are significantly different with greater accuracy for the biggest reward. The finding that accuracy improves going from 0p to the largest 50p reward level, is present in both groups (**Fig. S3B**).

Saccadic Reaction Time

There was no main effect of reward, ($F(2,78) = 0.38$, $P = 0.69$) or any reward by group interaction in reaction times, ($F(2,78) = 1.4$, $P = 0.25$). However, there was a main effect of group with elderly controls having significantly slower reactions times in general compared to young controls, ($F(1,39) = 26.7$, $P < 0.001$). (**Fig. S3C**).

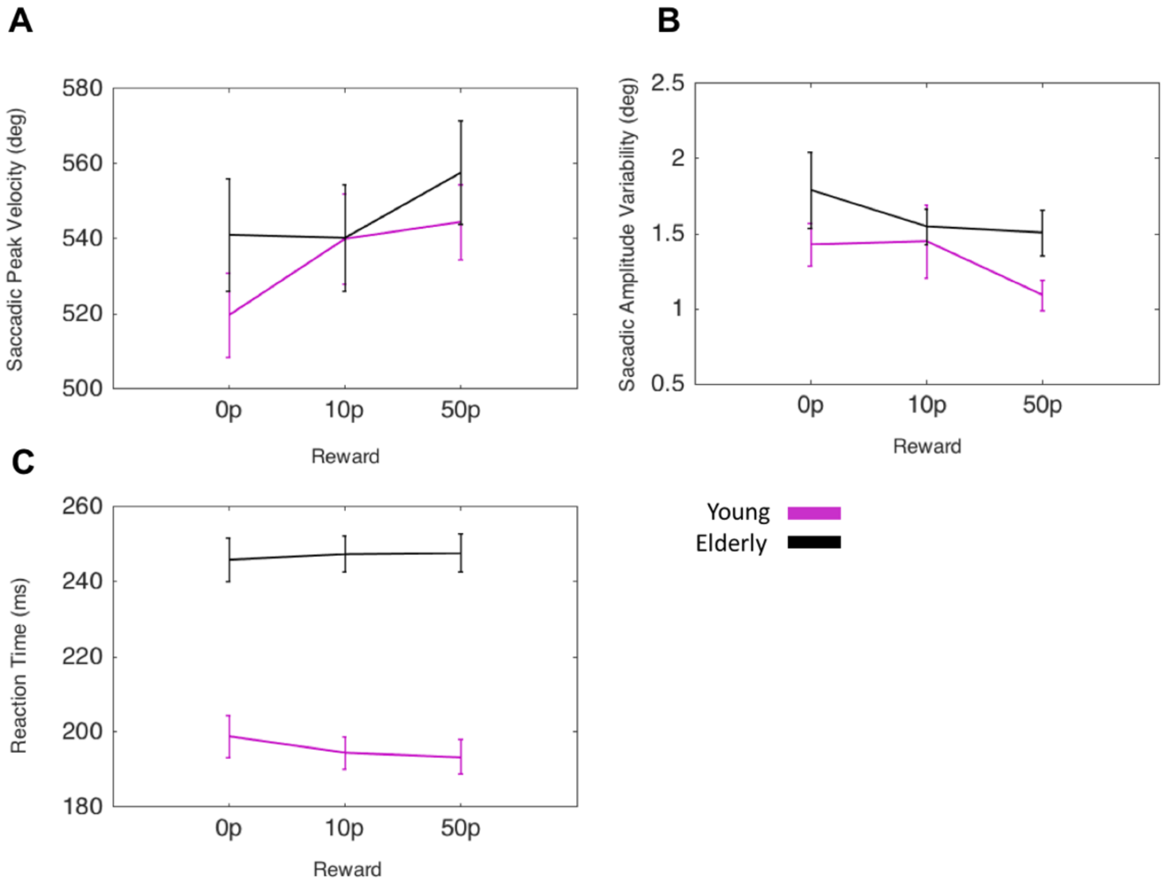

Figure S3 A-C. Comparison of oculomotor responses between young and elderly participants.

A. *Saccadic peak velocity increased for the largest level of reward on offer compared to 0p in both young (violet) and elderly groups (black). Younger participants had a larger change in their pupil response from 0p to any reward amount, whereas elderly participants only demonstrated an effect with the largest reward level on offer.*

B. *Saccadic accuracy as assessed by variability of saccade amplitude. No worsening of accuracy was seen for increasing levels of reward. There was an increase in accuracy when comparing between the 0p and 50p reward level both in the young (violet) and elderly (black) groups.*

C. *Reaction time for increasing reward levels did not differ between reward levels in either group but elderly participants had significantly slower reaction times overall.*

Parkinson's disease ON and OFF:

Saccadic peak velocity

When analyzed separately, PD patients ON dopamine show a significant increase in peak velocity for rewards of larger magnitude ($F(2,42) = 9.4$, $P < 0.001$), this was true

between each reward level ($p<0.03$). When in the OFF state, the extent to which peak velocity increased with reward was reduced, although there was still a main effect of reward ($F(2,42) = 8.0$, $P = 0.001$) the difference between each reward level was reduced with significance only between the 0p vs. 10p and 0p vs. 50p reward level ($p<0.02$) and no difference between the 10p vs. 50p ($p=0.241$). (**Fig. S4A,** ON – blue line, OFF – red line).

When comparing ON and OFF together in a combined repeated measures ANOVA, with drug and reward level, there was a main effect of reward but no statistically significant main effect of drug or interaction was present. Larger rewards lead to increasing speeds ($F(2,42) = 17.5$, $P < 0.00001$), and this was significant at every reward level comparison (0p vs 10p, 10p vs 50p and 0p vs 50p, $p<0.02$). (**Fig. S4A**).

Saccadic accuracy

When performing separate comparisons, a significant main effect of accuracy in PD ON or PD OFF was not present, ($F(2,42) = 0.45$, $P = 0.643$ and $F(2,42) = 3.0$, $P = 0.06$ respectively). Interestingly, by performing a 2x3 repeated measures ANOVA comparing accuracy between reward levels and drug state, a significant main effect of drug emerged ($F(1,21) = 7.5$, $P = 0.012$) with those ON dopamine having on average worse accuracy (increased saccadic variability) compared to those OFF. (**Fig. S4B**).

Saccadic reaction times

There was no significant main effect of reaction time in PD ON ($F(2,42) = 1.2$, $P = 0.313$) or PD OFF ($F(2,42) = 2.6$, $P = 0.09$). When comparing drug and reward effects on

reaction time together there was a trend for faster reaction time with increasing reward levels (F(2,42) =2.6, P = 0.09) and a main effect of drug (F(1,21) =14.9, P = 0.001) demonstrating that when OFF dopamine, reaction times in general were actually faster than when ON. Perhaps surprisingly, being ON dopamine medication seemed to increase saccadic latency in general. (**Fig. S4C**).

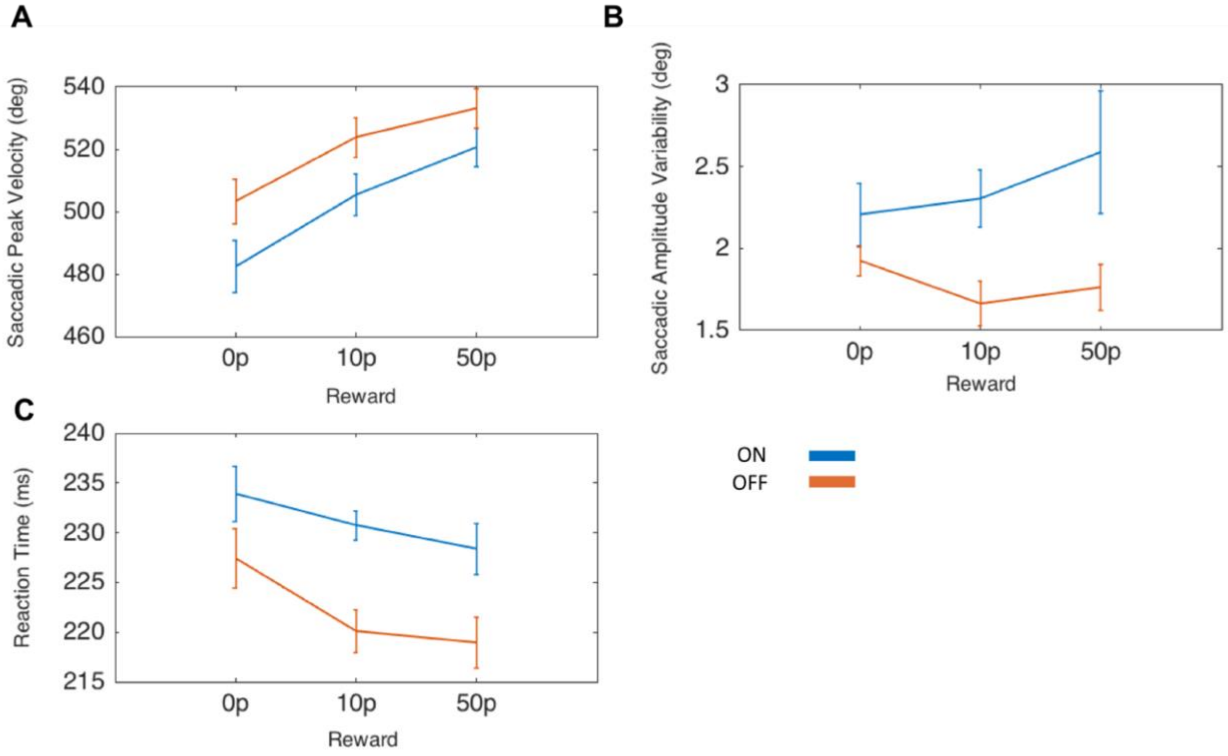

Figure S4 A-C. ***Comparison of oculomotor responses between PD patients ON and OFF dopamine.***

A. *Saccadic peak velocity increased for the increasing levels of reward on offer both when ON (blue) and OFF (red) dopamine.*

B. *Saccadic accuracy as assessed by variability of saccade amplitude. No worsening of accuracy for increasing levels of reward when ON (blue) or OFF (red) was detected. However, when ON dopamine the variability of saccades seemed to be greater overall when compared to OFF.*

C. *Reaction time for increasing reward levels did not differ between reward levels in either group but surprisingly patients ON (blue) dopamine had significantly slower reaction times overall compared to OFF (red).*

Parkinson's disease versus elderly controls:

Saccadic peak velocity

Performing a repeated measures ANOVA on the peak velocities for the 3 reward levels and a between groups comparison between elderly and PD patients ON revealed a main effect of reward ($F(2, 76) = 12.7$, $P < 0.0001$), and interestingly a trend towards a group by reward interaction ($F(2, 76) = 2.9$, $P = 0.06$). Exploring this trend suggests that PD patients ON dopamine have a greater response to reward in terms of saccadic peak velocity (steeper slope) compared to elderly controls. Although this did not reach statistical significance, potential explanations could include a trend for hyper-responsiveness to reward while being on dopamine therapy. (**Fig. S5A** – blue and black lines).

PD patients OFF compared to elderly controls also demonstrated a main effect of reward ($F(2, 76) = 11.5$, $P < 0.0001$), and a less strong trend towards a group by reward interaction ($F(2, 76) = 2.5$, $P = 0.091$), (**Fig. S5A** – red and black lines). There was no main effect of group between elderly controls and PD patients both ON or OFF.

Saccadic accuracy

No main effects of reward or any interaction between elderly controls and PD patients when ON dopamine were present. There was however a worse overall effect of accuracy in PD patients ON dopamine with a main effect of group present ($F(1, 38) = 4.6$, $P = 0.038$), (**Fig. S5B**). This was abolished when OFF dopamine, with no main effect of group surviving comparison and no interaction present. A weak trend towards a significant main

effect of reward ($F(2, 76) = 2.6$, $P = 0.08$) was seen when comparing PD OFF and elderly controls, with a trend towards more accurate and less variable saccades for larger rewards on offer.

Saccadic Reaction Time

There were no main effects of group, reward or any significant interaction when ON dopamine compared to elderly controls, (**Fig. S5C**). Perhaps surprisingly, when OFF dopamine, reaction times in PD were significantly faster in general compared to elderly controls with a main effect of group being present ($F(1, 38) = 4.5$, $P = 0.04$). There were no main effects of reward and no group by reward interaction between elderly controls and PD OFF.

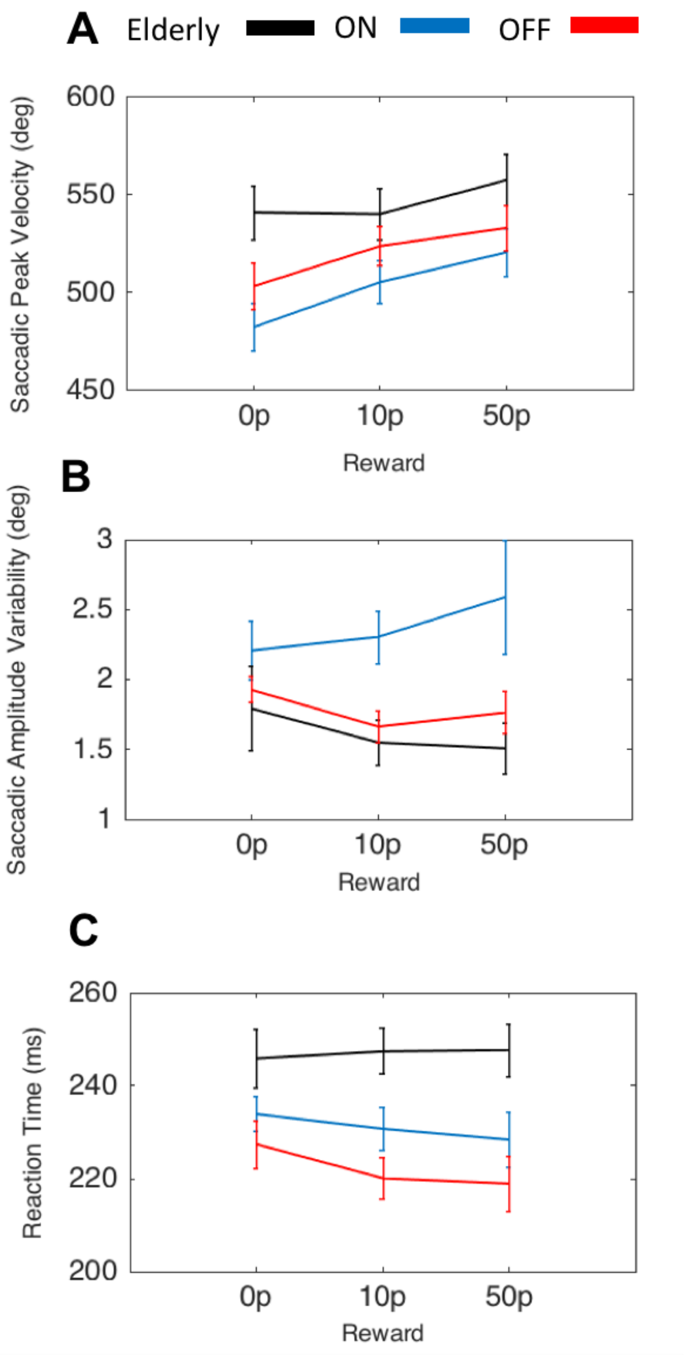

Figure S5 A-C. Oculomotor properties in PD patients ON and OFF dopamine and elderly controls.

- ***A.*** *Faster saccades for larger rewards were present in PD ON, PD OFF and elderly controls, no significant group differences were present. A trend towards a steeper slope in PD patients ON dopamine compared to elderly controls was observed, p=0.06.*
- ***B.*** *PD patients ON had increased saccadic variability overall compared to elderly controls. When OFF dopamine there were no differences in saccadic accuracy between patients and elderly controls, although a trend towards more accurate saccades was seen in elderly participants and PD patients when OFF, p=0.08.*

C. *No differences were present between PD patients ON dopamine and elderly controls in saccadic reaction time. When OFF dopamine, PD patients had faster reaction times in general compared to elderly controls, however no significant effect of reward was present.*

When looking at the effect of dopamine on saccadic variability and latency, it seems that reaction time becomes slower and accuracy worsens while ON dopamine. This worsening in performance is surprising and may reflect increased dopamine neuronal activity in the substantia nigra, which has been shown to impair attention and delay responsivity in rats (Boekhoudt *et al.*, 2016). Therefore, this nigrostriatal pathway may be connected to attentional processes and stimulation of this pathway through dopaminergic medication in our study may have resulted in impaired reaction time.

References:

Boekhoudt L, Voets ES, Flores-Dourojeanni JP, Luijendijk MC, Vanderschuren LJ, Adan RA. Chemogenetic Activation of Midbrain Dopamine Neurons Affects Attention, but not Impulsivity, in the Five-Choice Serial Reaction Time-Task in Rats. Neuropsychopharmacology 2016; 42: 1–11.
